# Correction: Hou et al. Emerging Role of Vitamins D and K in Modulating Uremic Vascular Calcification: The Aspect of Passive Calcification. *Nutrients 2019, 11(1):152*

**DOI:** 10.3390/nu11030567

**Published:** 2019-03-06

**Authors:** Yi-Chou Hou, Chien-Lin Lu, Cai-Mei Zheng, Ruei-Ming Chen, Yuh-Feng Lin, Wen-Chih Liu, Tzung-Hai Yen, Remy Chen, Kuo-Cheng Lu

**Affiliations:** 1 Department of Internal Medicine, Cardinal Tien Hospital, School of Medicine, Fu-Jen Catholic University, New Taipei City 23148, Taiwan; athletics910@gmail.com; 2 College of Medicine, Fu-Jen Catholic University, New Taipei City 24205, Taiwan; janlin0123@gmail.com; 3 Graduate Institute of Clinical Medicine, College of Medicine, Taipei Medical University, Taipei 11031 Taiwan; 11044@s.tmu.edu.tw (C.-M.Z.); linyf@shh.org.tw (Y.-F.L.); wayneliu55@gmail.com (W.-C.L.); 4 Division of Nephrology, Department of Medicine, Fu Jen Catholic University Hospital, School of Medicine, Fu Jen Catholic University, New Taipei City 24205, Taiwan; 5 Division of Nephrology, Department of Internal Medicine, Shuang Ho Hospital, Taipei Medical University, New Taipei City 23561, Taiwan; 6 Graduate Institute of Medical Sciences, College of Medicine, Research Center of Cancer Translational Medicine, Taipei Medical University, Taipei 11031, Taiwan; rmchen@tmu.edu.tw; 7 Anesthesiology and Health Policy Research Center, Taipei Medical University Hospital, Taipei 11031, Taiwan; 8 Brain Disease Research Center, Wan-Fang Hospital, Taipei Medical University, Taipei 11031, Taiwan; 9 Division of Nephrology, Department of Internal Medicine, Tri-Service General Hospital, Taipei 11490, Taiwan; 10 Division of Nephrology, Department of Internal Medicine, Tungs’ Taichung MetroHarbor Hospital, Taichung City 435, Taiwan; 11 Department of Nephrology, Chang Gung Memorial Hospital and College of Medicine, Chang Gung University, Taoyuan City 33305, Taiwan; m19570@adm.cgmh.org.tw; 12 Kidney Research Center, Chang Gung Memorial Hospital, Taoyuan City 33305, Taiwan; 13 Center for Tissue Engineering, Chang Gung Memorial Hospital, Taoyuan City 33305, Taiwan; 14 Kidney Dialysis Center, Kamifukuoka General Hospital, Saitama 356, Japan; remyneko@yahoo.co.jp

The authors wish to make the following changes to their paper (Hou et al. [1]). 

1. The concepts of our article are based on the results of important studies, and the authors of 2 articles suggested to us modifying the concepts in order to match their study results. 

**Published version:**

A. Introduction (p2/14): Furthermore, vitamin K supplements were found to be crucial for stabilizing CPPS and MVs in patients with CKD [13]. Through this review, we would to elucidate the relationship between vitamin K deficiency and uremic calcification, as well as the role of vitamin supplements in the prevention of uremic vascular calcification. 

B. Vitamin D Supplementation as a Potential Target for Salvaging Uncarboxylated MGP (p9/14): Van Ballegooijen et al. provided evidence that vitamin D deficiency (< 50 mmol/L), along with vitamin K deficiency, predicts higher risk of hypertension in the Netherlands [7].

**Revised version:**

A. Introduction (p2/14): Supplement of calcification inhibitors such as carboxylated GRP should play a role in alleviating systemic calcification in CKD patients [13]. Since vitamin K governs gamma-carboxylation of Gla matrix proteins, we would to elucidate the relationship between vitamin K deficiency and uremic calcification, as well as the role of vitamin supplements in the prevention of uremic vascular calcification.

B. Vitamin D Supplementation as a Potential Target for Salvaging Uncarboxylated MGP (p9/14): Van Ballegooijen et al. provided evidence that vitamin D deficiency (< 50 mmol/L), along with vitamin K deficiency, was associated with higher systolic and diastolic blood pressure [82].

2. The references number were missing in the published version. After correction, we inserted the appropriate references at the proper sections. The references number increased from 83 to 90. The newly inserted references are in the revised version listed in the following sections:

**Inserted References**

Ref. 47. Van Ballegooijen, A.J.; Beulens, J.W. The Role of Vitamin K Status in Cardiovascular Health: Evidence from Observational and Clinical Studies. Curr. Nutr. Rep. 2017, 6, 197–205. (p6/14) 

Ref. 64. Holden, R.M.; Booth, S.L.; Day, A.G.; Clase, C.M.; Zimmerman, D.; Moist, L.; Shea, M.K.; McCabe, K.M.; Jamal, S.A.; Tobe, S., et al. Inhibiting the progression of arterial calcification with vitamin K in HemoDialysis patients (iPACK-HD) trial: rationale and study design for a randomized trial of vitamin K in patients with end stage kidney disease. Canadian J Kidney Health Disease 2015, 2, 17. (p7/14)

Ref. 74. Fraser, J.D.; Price, P.A. Induction of matrix Gla protein synthesis during prolonged 1,25-dihydroxyvitamin D3 treatment of osteosarcoma cells. Calcif. Tissue Int. 1990, 46, 270–279. (p7/14)

Ref. 79. Seyama, Y.; Horiuch, M.; Hayashi, M.; Kanke, Y. Effect of vitamin K2 on experimental calcinosis induced by vitamin D2 in rat soft tissue. Int. J. Vitam. Nutr. Res. 1996, 66, 36–38. (p8/14)

Ref. 80. Bostrom, K.I. Cell differentiation in vascular calcification. Z Kardiol 2000, 89, 69–74. (p8/14)

Ref. 81. Farzaneh-Far, A.; Weissberg, P.L.; Proudfoot, D.; Shanahan, C.M. Transcriptional regulation of matrix gla protein. Z Kardiol 2001, 90, 38–42. (p8/14)

Ref. 82. van Ballegooijen, A.J.; Cepelis, A.; Visser, M.; Brouwer, I.A.; van Schoor, N.M.; Beulens, J.W. Joint Association of Low Vitamin D and Vitamin K Status With Blood Pressure and Hypertension. Hypertension 2017, 69, 1165–1172. (p9/14)

No other references are modified. No further adjustment of the order of references were made. The mistakes were unintentional and do not impact the main findings of our paper. The authors would like to apologize for any inconvenience caused to the readers by this change. The changes do not affect the results and we apologize for the oversight.
